# Parental occupations, educational levels, and income and prevalence of dental caries in 3-year-old Japanese children

**DOI:** 10.1186/s12199-017-0688-6

**Published:** 2017-12-13

**Authors:** Hiromasa Kato, Keiko Tanaka, Ken Shimizu, Chisato Nagata, Shinya Furukawa, Masashi Arakawa, Yoshihiro Miyake

**Affiliations:** 1 Department of Epidemiology and Preventive Medicine, Ehime University Graduate School of Medicine, Shitsukawa, Toon, Ehime, 791-0295 Japan; 2 Kato Dental Clinic, Ehime, Japan; 3 Epidemiology and Medical Statistics Unit, Translational Research Center, Ehime University Hospital, Ehime, Japan; 4 Department of Epidemiology and Preventive Medicine, Gifu University Graduate School of Medicine, Gifu, Japan; 5 Department of Epidemiology and Preventive Medicine, Ehime University Graduate School of Medicine, Shitsukawa, Toon, Ehime Japan; 6 Health Tourism Research Fields, Graduate School of Tourism Sciences, University of the Ryukyus, Okinawa, Japan

**Keywords:** Cross-sectional studies, Dental caries, Education, Employment, Income, Occupations, Socioeconomic factors

## Abstract

**Background:**

Most studies have investigated the association between parental socioeconomic factors and dental caries in children based on educational and income levels; studies focusing on parental occupation, however, have been relatively limited. This cross-sectional study examined the associations between parental occupations and levels of education and household income and the prevalence of dental caries in Japanese children aged 3 years.

**Methods:**

Study subjects were 6315 children. Oral examination results were obtained from the parents or guardians, who transcribed the information recorded by medical staff at a public health center from their maternal and child health handbooks to our self-administered questionnaire. Children were classified as having dental caries if one or more primary teeth had decayed or had been filled. Adjustment was made for sex, age, region of residence, breastfeeding duration, between-meal snack frequency, toothbrushing frequency, use of fluoride, regular dental check-ups, maternal smoking during pregnancy, and living with at least one household smoker.

**Results:**

The prevalence of dental caries was 14.7%. Compared with having an unemployed father, having a father employed in professional and engineering, clerical, sales, security, or manufacturing process was significantly associated with a lower prevalence of dental caries. Compared with having an unemployed mother, having a mother employed in professional and engineering or service was significantly inversely associated with the prevalence of dental caries. Significant inverse associations were observed between parental levels of education and household income and the prevalence of dental caries.

**Conclusions:**

The findings of our study suggest that parental occupation affects the prevalence of dental caries in children. We confirm that higher levels of parental education and household income decreased the prevalence of dental caries.

## Background

Dental caries is one of the most prevalent chronic diseases worldwide [1, 2]. It is a multifactorial disease involving a complex interplay of microbial, genetic, biochemical, and social factors [1].

Most studies reporting risk factors for dental caries in children have focused on biological and behavioral factors, such as colonization of cariogenic microorganisms, the use of fluoride, dietary habits, and oral health behaviors [3, 4]. Because socioeconomic factors are likely to influence the prevalence of dental caries in children through their effects on oral health practices and parental oral health knowledge and attitudes, they are gaining increased attention in recent years in studies on the prevention and control of dental caries [5–8].

Two recent systematic reviews, conducted in 2012 [5] and 2016 [7], revealed that parental socioeconomic status (SES) was inversely associated with dental caries in children aged 0 to 6 years [5] and in children aged 6 to 12 years [7]. Another recent meta-analysis showed an inverse association between SES and dental caries among adults and children [6]. Of the studies included in these two systematic reviews and one meta-analysis, the majority estimated parental SES based on educational and income levels, but studies based on parental occupation have been relatively limited. Thus, in addition to parental education and income, it is necessary to accumulate further evidence of the association between parental occupation and caries in children.

The purpose of the current cross-sectional study was to examine the associations between parental occupation and levels of education, and household income and the prevalence of dental caries, using data from the Kyushu Okinawa Child Health Study (KOCHS).

## Methods

### Study subjects

In Japan, as a provision of the Maternal and Child Health Law, when a child is between 36 and 47 months of age, the municipality where the child resides performs a physical examination, which includes an oral examination, anthropometric measurements of height and weight, and an interview with parents or guardians about the child’s health status. Eligible subjects for the KOCHS were children aged 3 years who underwent a physical examination at a public health center in one of the 45 municipalities in six prefectures on Kyushu Island in southern Japan or Okinawa Prefecture, an island chain in the southwest of Japan, between May 2012 and March 2014. Of the 68,527 eligible subjects, we provided 62,449 parents or guardians with a structured self-administered questionnaire and a postage-paid addressed return envelope. Ultimately, a total of 6576 parents or guardians gave their informed written consent, answered the questionnaires, and mailed these materials to the data management center (participation rate = 9.6%). Our research technicians completed missing answers and/or illogical data by telephone interview with individual parents or guardians. A total of 261 children with incomplete data on the variables under study were excluded from the current study, leaving data on 6315 available for analysis (9.2% of the 68,527 eligible children). The ethics committees of the Faculty of Medicine, Fukuoka University and Ehime University Graduate School of Medicine approved the KOCHS. The STROBE (Strengthening the Reporting of Observational studies in Epidemiology) guidelines were followed.

### Measurements

At the time of the physical examination, the presence of dental caries was assessed by visual examination by a dentist under artificial light with a dental mirror. Radiographs were not taken. Data from the dental examination were recorded at the tooth level in each child’s maternal and child health handbook, which is provided by the municipality during each pregnancy and which included information on prenatal checkups as well as postnatal health conditions of both the mother and baby as well as the growth of the child. In the KOCHS, the parents or guardians were required to transcribe the data on the dental examination from the maternal and child health handbook to our self-administered questionnaire. We classified children as having dental caries if one or more primary teeth had decayed or had been filled because the reasons for missing primary teeth were not identified in the present study. Information on paternal and maternal employment status and type of job during the year prior to the conception of the child, paternal and maternal educational levels, household income, sex, breastfeeding duration, dental health behavior (such as toothbrushing frequency, use of fluoride, and pattern of dental care), between-meal snack frequency, maternal smoking during pregnancy, and smoking habits of the adult household members were obtained from a self-administered questionnaire. Use of fluoride was defined as positive if children were reported to use fluoride toothpaste at home or to receive the application of topical fluoride products at a dental clinic or a public health center. Living with at least one household smoker was defined as positive if the child had lived with at least one smoker at any point up to the survey.

### Statistical analysis

Parental occupation was classified according to the Japan Standard Occupational Classification and stratified into 12 major groups: administrative and managerial; professional and engineering; clerical; sales; service; security; agriculture, forestry and fishery; manufacturing process; transport and machine operation; construction and mining; carrying, clearing, packaging and related; and not classified by occupation. Paternal and maternal educational levels were classified into three categories: < 13, 13–14, and ≥ 15 years; these levels are equivalent to high school, junior college or vocational/technical school, and university or higher, respectively. Household income was classified into three categories (< 4,000,000, 4,000,000–5,999,999, and ≥ 6,000,000 yen/year).

Sex, age, region of residence, breastfeeding duration, between-meal snack frequency, toothbrushing frequency, use of fluoride, regular dental check-ups, maternal smoking during pregnancy, and living with at least one household smoker were selected as a priori potential confounding factors. Region of residence was classified into three categories (Fukuoka City, municipalities in Kyushu other than Fukuoka City, and municipalities in Okinawa Prefecture), breastfeeding duration into two (< 12 and ≥ 12 months), between-meal snack frequency into three (< 1, 1, and ≥ 2 times/day), toothbrushing frequency into three (< 1, 1, and ≥ 2 times/day), use of fluoride into two (yes and no), regular dental check-ups into two (yes and no), maternal smoking during pregnancy into two (yes and no), and living with at least one household smoker into two (ever and never). Logistic regression analysis was performed to estimate the crude odds ratios (ORs) and their 95% confidence intervals (CIs) for dental caries according to parental occupations and educational levels and household income. Additionally, multiple logistic regression analysis was conducted to control for potential confounders. All statistical analyses were performed using the SAS software package version 9.4 (SAS Institute, Inc., Cary, NC, USA).

## Results

Of the 6315 children, 927 (14.7%) had experienced dental caries. The mean number of teeth that were decayed or filled for all subjects and for subjects who had dental caries was 0.5 and 3.2, respectively.

Table 1 shows the characteristics of the study subjects. Approximately 30% of children were breastfed for less than 12 months. Approximately 46% of the subjects were provided with between-meal snacks two or more times per day and reported two or more times daily toothbrushing. Approximately 92% of children used fluoride agents. Approximately 56% of children received regular dental check-ups. Approximately 9 and 42% of children had been exposed to maternal smoking during pregnancy and had ever lived with at least one household smoker, respectively.

**Table 1:** Distribution of selected characteristics in 6315 children aged 3 years

Variable	*n* (%) or mean ± SD
Male sex	3210 (50.8)
Age, months, mean ± SD	38.7 ± 2.6
Region of residence	
Fukuoka City	2529 (40.1)
Municipalities on Kyushu other than Fukuoka City	3021 (47.8)
Municipalities in Okinawa Prefecture	765 (12.1)
Breastfeeding duration (months)	
< 12	1920 (30.4)
≥ 12	4395 (69.6)
Between-meal snack frequency (times/day)	
< 1	1357 (21.5)
1	2077 (32.9)
≥ 2	2881 (45.6)
Toothbrushing frequency (times/day)	
< 1	752 (11.9)
1	2670 (42.3)
≥ 2	2893 (45.8)
Use of fluoride	
No	498 (7.9)
Yes	5817 (92.1)
Regular dental check-ups	
No	2759 (43.7)
Yes	3556 (56.3)
Maternal smoking during pregnancy	
No	5721 (90.6)
Yes	594 (9.4)
Living with at least one household smoker	
Never	3675 (58.2)
Ever	2640 (41.8)

*SD* standard deviation

Table 2 gives crude and adjusted ORs and their 95% CIs for dental caries in relation to parental occupation. After adjustment for the confounding factors under study, compared with having an unemployed father, having a father who worked in professional and engineering, clerical, sales, security, or manufacturing process was associated with a lower prevalence of dental caries in children: adjusted ORs (95% CIs) were 0.60 (0.38–0.97), 0.60 (0.37–0.99), 0.61 (0.38–0.98), 0.55 (0.31–0.96), and 0.60 (0.37–0.998), respectively. No associations were observed between having a father who worked in administrative and managerial, service, agriculture, forestry and fishery, transport and machine operation, construction and mining, carrying, cleaning, packaging, and related, or not classified by occupation and the prevalence of caries in children. Compared with having an unemployed mother, having a mother who worked in professional and engineering or service was independently associated with a lower prevalence of dental caries in children: adjusted ORs (95% CIs) were 0.69 (0.56–0.84) and 0.76 (0.58–0.98), respectively. No associations were observed between having a mother who worked in administrative and managerial, clerical, sales, manufacturing process, or others and the prevalence of caries in children.

**Table 2:** ORs and 95% CIs for dental caries in relation to parental occupation in 6315 Japanese children aged 3 years

	Prevalence	Crude OR (95% CI)	Adjusted OR (95% CI)^b^
Paternal occupation			
Unemployed	28/121 (23.1%)	1.00	1.00
Administrative and managerial	102/621 (16.4%)	0.65 (0.41–1.06)	0.80 (0.50–1.32)
Professional and engineering	179/1418 (12.6%)	0.48 (0.31–0.77)	0.60 (0.38–0.97)
Clerical	84/659 (12.8%)	0.49 (0.30–0.79)	0.60 (0.37–0.99)
Sales	150/1159 (12.9%)	0.49 (0.32–0.79)	0.61 (0.38–0.98)
Service	82/498 (16.5%)	0.66 (0.41–1.08)	0.70 (0.43–1.17)
Security	37/286 (12.9%)	0.49 (0.29–0.86)	0.55 (0.31–0.96)
Agriculture, forestry, and fishery	15/86 (17.4%)	0.70 (0.34–1.40)	0.70 (0.34–1.42)
Manufacturing process	84/614 (13.7%)	0.53 (0.33–0.86)	0.60 (0.37–0.998)
Transport and machine operation	33/172 (19.2%)	0.79 (0.45–1.40)	0.89 (0.50–1.59)
Construction and mining	90/437 (20.6%)	0.86 (0.54–1.41)	0.90 (0.56–1.50)
Carrying, cleaning, packaging, and related	39/231 (16.9%)	0.68 (0.39–1.17)	0.72 (0.41–1.27)
Not classified by occupation	4/13 (30.8%)	1.48 (0.38–4.91)	1.68 (0.41–5.84)
Maternal occupation			
Unemployed	344/2183 (15.8%)	1.00	1.00
Administrative and managerial	9/39 (23.1%)	1.60 (0.71–3.27)	1.65 (0.72–2.43)
Professional and engineering	172/1449 (11.9%)	0.72 (0.59–0.88)	0.69 (0.56–0.84)
Clerical	189/1347 (14.0%)	0.87 (0.72–1.06)	0.82 (0.68–1.001)
Sales	86/479 (18.0%)	1.17 (0.90–1.51)	1.08 (0.82–1.40)
Service	88/620 (14.2%)	0.88 (0.68–1.13)	0.76 (0.58–0.98)
Manufacturing process	19/103 (18.5%)	1.21 (0.71–1.97)	0.99 (0.57–1.64)
Others^a^	20/95 (21.1%)	1.43 (0.84–2.32)	1.34 (0.78–2.21)

*CI* Confidence interval

*OR* Odds ratio

^a^Others included security, agriculture, forestry and fishery, transport and machine operation, construction and mining, carrying, cleaning, packaging, and related and not classified by occupation

^b^Adjustment for sex, age, region of residence, breastfeeding duration, between-meal snack frequency, toothbrushing frequency, use of fluoride, regular dental check-ups, maternal smoking during pregnancy, and living with at least one household smoker

Table 3 presents crude and adjusted ORs and their 95% CIs for the relationship between paternal and maternal educational levels and household income and the prevalence of dental caries in children. Compared with less than 13 years of paternal and maternal education, 13–14 years and 15 or more years of paternal and maternal education were independently inversely associated with the prevalence of dental caries in children. The test for trend was also statistically significant (*P* for trend in paternal and maternal educational levels were < 0.0001 and < 0.0001, respectively). Compared with the lowest household income category, the middle and highest household income categories were significantly associated with a lower prevalence of dental caries in children. A stepwise trend by income level was found (*P* for trend < 0.0001).

**Table 3:** ORs and 95% CIs for dental caries in relation to parental educational levels and household income in 6315 Japanese children aged 3 years

	Prevalence	Crude OR (95% CI)	Adjusted OR (95% CI)^a^
Paternal education (years)			
< 13	374/1948 (19.2%)	1.00	1.00
13–14	134/918 (14.6%)	0.72 (0.58–0.89)	0.75 (0.60–0.93)
≥ 14	419/3449 (12.2%)	0.58 (0.50–0.68)	0.69 (0.59–0.82)
*P* for trend		< 0.0001	< 0.0001
Maternal education (years)			
< 13	304/1505 (20.2%)	1.00	1.00
13–14	294/2118 (13.9%)	0.64 (0.53–0.76)	0.72 (0.60–0.87)
≥ 14	329/2692 (12.2%)	0.55 (0.46–0.65)	0.64 (0.53–0.77)
*P* for trend		< 0.0001	< 0.0001
Household income (yen/year)			
< 4,000,000	371/1969 (18.8%)	1.00	1.00
4,000,000–5,999,999	312/2147 (14.5%)	0.73 (0.62–0.86)	0.84 (0.71–0.995)
≥ 6,000,000	244/2199 (11.1%)	0.54 (0.45–0.64)	0.66 (0.55–0.79)
*P* for trend		< 0.0001	< 0.0001

*CI* confidence interval

*OR* odds ratio

^a^Adjustment for sex, age, region of residence, breastfeeding duration, between-meal snack frequency, toothbrushing frequency, use of fluoride, regular dental check-ups, maternal smoking during pregnancy, and living with at least one household smoker

When parental occupations and educational levels and household income were simultaneously taken into account, inverse associations with the prevalence of dental caries in children remained for the highest category of paternal educational level, the highest and middle categories of maternal educational level, the highest category of household income, and having a mother who worked in professional and engineering and service: the additionally adjusted ORs (95% CIs) were 0.80 (0.66–0.97), 0.77 (0.63–0.95), 0.80 (0.66–0.97), 0.73 (0.59–0.89), 0.81 (0.65–0.998), and 0.73 (0.55–0.95).

## Discussion

There is limited evidence regarding the association between parental occupation and dental caries in young children [9–16]. In a 4-year prospective study among Scottish children from ages 1 to 4, compared with unemployment, parental employment was significantly associated with lower rate of caries increment [9]. A cross-sectional study in Switzerland showed that parental occupations with higher professional level were significantly inversely associated with the prevalence of caries in children aged 36 to 71 months [10]. Among 7-year-old Belgian children, skilled non-manual, skilled manual, semi-skilled, and unskilled parental occupations as well as managerial, skilled non-manual, skilled manual, semi-skilled, and unskilled maternal occupations as well as unemployment of either parent were associated with a higher prevalence of dental caries [16]. On the other hand, no association was observed between maternal employment and dental caries in Japanese [11], Brazilian [12], South African [13], Mongolian [14], and African-American [15] preschool children. Most of the studies that have examined the association between parental occupation and dental caries have used a crude measurement of employment status, such as employment vs. unemployment. In studies using such crude classifications, the category of employment includes a wide range of occupations with different characteristics. This type of classification may not be sufficiently informative about specific occupations. In the present study, we assessed the parental occupations-dental caries association using a more detailed classification.

Our findings that parental educational levels and household income were inversely associated with the prevalence of dental caries are consistent with the previously reported findings that lower paternal and/or maternal educational levels [9–13, 17–23] and household income [10, 12, 23–25] were significantly positively associated with dental caries in young children, but differ from other previous results showing null findings of family income [11, 13, 17] or parental education [15, 26]. A recent systematic review concluded that lower family income and parental education were associated with higher risk of dental caries in children aged 0–6 years [5].

We do not have a definitive explanation regarding the mechanisms underlying the observed association between parental occupations, educational levels, and family income and the prevalence of dental caries. Parental SES might influence dental caries in children through oral health knowledge and practices [27]. A German cross-sectional study demonstrated that parents with high and middle SES as determined based on parental education, vocational training, and occupational status were significantly more likely to start taking care of their children’s teeth before the second year of life and also to help them brush their teeth between the third and sixth year of life [28].

After mutual adjustment, independent inverse associations remained between the highest category of paternal educational level, the highest and middle categories of maternal educational level, the highest category of household income, and having a mother who worked in a professional and engineering or service occupation and the prevalence of dental caries in children. Thus, maternal factors seem to have a greater impact than paternal factors do on dental caries prevalence in our population. This may be because mothers typically provide a proportionally larger degree of child nurturing.

This study has several methodological strengths. The sample size in the current study was larger than those of most studies investigating the association between SES and dental caries. Therefore, we could evaluate the effects of parental occupation on dental caries using a detailed classification of occupations. Data on dental caries were obtained from dental examinations by dentists. We could control for comprehensive potential confounders.

This study has some limitations, and the results therefore need to be interpreted carefully. Some selection bias is inevitable as only 9.2% of all eligible subjects were included in this analysis. Our subjects were not representative of Japanese children in the general population. Therefore, it would be difficult to generalize the present findings. In fact, parental education levels in our study were higher than that in the general population [29]. On the other hand, according to the Report on the Survey of Dental Diseases in 2011, the prevalence of dental caries in the sample of 3-year-old Japanese children was 25.0% [30], while the prevalence of dental caries in our study subjects was only 14.7%.

The fact that this is a cross-sectional study precludes the establishment of a causal relationship between parental occupations and educational levels and household income and dental caries. In the present study, the data on dental caries were gathered during routine examinations by dentists at public health centers. Examiners did not receive specific training aimed at standardization of the procedure. Additionally, no reliability assessment of measurements was carried out in the present study. Thus, it is unknown whether intra- and inter-examiner agreement was established. In the present study, parents or guardians transcribed the data on dental examinations from their maternal and child health handbook to our self-administered questionnaire; therefore, we cannot exclude the possibility that transcription errors occurred. The outcome misclassification is unlikely to differ across categories of exposures, however. The non-differential outcome misclassification leads to an underestimation. Moreover, in the present study, our outcome was dichotomous and thus did not provide data on the severity of the disease.

We used self-reported data on socioeconomic factors. Some participants might have reported a higher education level or income than they actually possessed, which could have affected the results. Although we adjusted for several potential confounding factors, other unmeasured factors could have influenced our findings and might even explain the inverse associations identified in the current study.

## Conclusions

Our study provides evidence that having a father who works in professional and engineering, clerical, sales, security, and manufacturing process and having a mother who works in professional and engineering and service are associated with a lower prevalence of dental caries in children. We confirm significant inverse associations between parental educational levels and household income and the prevalence of dental caries. Although underlying mechanisms of observed associations are remaining unclear, for instance, more attention may have to be payed children having parents with certain occupations and low educational levels and household income in order to prevent childhood dental caries.

Despite the numerous limitations, our study provides valuable insights into the effects of parental occupations on dental caries in children. Further studies are needed to clarify the roles of parental socioeconomic factors on dental caries as well as the underlying mechanisms involved.

## Abbreviations

CI: Confidence interval
KOCHS: Kyushu Okinawa Child Health Study
OR: Odds ratio
SES: Socioeconomic status
